# Serum Reactive Oxygen Metabolite Levels Predict Severe Exacerbations of Asthma

**DOI:** 10.1371/journal.pone.0164948

**Published:** 2016-10-24

**Authors:** Keitaro Nakamoto, Masato Watanabe, Mitsuru Sada, Toshiya Inui, Masuo Nakamura, Kojiro Honda, Hiroo Wada, Yu Mikami, Hirotaka Matsuzaki, Masafumi Horie, Satoshi Noguchi, Yasuhiro Yamauchi, Hikari Koyama, Toshiyuki Kogane, Tadashi Kohyama, Hajime Takizawa

**Affiliations:** 1 Department of Respiratory Medicine, Kyorin University School of Medicine, Tokyo, Japan; 2 Department of Respiratory Medicine, Graduate School of Medicine, The University of Tokyo, Tokyo, Japan; 3 Department of Internal medicine, Teikyo University Mizonokuchi Hospital, Kanagawa, Japan; Kyoto Daigaku, JAPAN

## Abstract

**Background and Purpose:**

Bronchial asthma (BA) is a chronic airway disease characterized by airway hyperresponsiveness and remodeling, which are intimately linked to chronic airway inflammation. Reactive oxygen species (ROS) such as hydrogen peroxide are generated by inflammatory cells that are involved in the pathogenesis of BA. However, the role of ROS in the management of BA patients is not yet clear. We attempted to determine the role of ROS as a biomarker in the clinical setting of BA.

**Subjects and Methods:**

We enrolled patients with BA from 2013 through 2015 and studied the degrees of asthma control, anti-asthma treatment, pulmonary function test results, fractional exhaled nitric oxide (FeNO), serum reactive oxygen metabolite (ROM) levels, and serum levels of interleukin (IL)-6 and IL-8.

**Results:**

We recruited 110 patients with BA. Serum ROM levels correlated with white blood cell (WBC) count (rs = 0.273, *p* = 0.004), neutrophil count (rs = 0.235, *p* = 0.014), CRP (rs = 0.403, *p* < 0.001), and IL-6 (rs = 0.339, *p* < 0.001). Serum ROM levels and IL-8 and CRP levels negatively correlated with %FEV_1_ (rs = -0.240, *p* = 0.012, rs = -0.362, *p* < 0.001, rs = -0.197, *p* = 0.039, respectively). Serum ROM levels were significantly higher in patients who experienced severe exacerbation within 3 months than in patients who did not (339 [302–381] vs. 376 [352–414] CARR U, *p* < 0.025). Receiver-operating characteristics analysis showed that ROM levels correlated significantly with the occurrence of severe exacerbation (area under the curve: 0.699, 95% CI: 0.597–0.801, *p* = 0.025).

**Conclusions:**

Serum levels of ROM were significantly associated with the degrees of airway obstruction, WBC counts, neutrophil counts, IL-6, and severe exacerbations. This biomarker may be useful in predicting severe exacerbations of BA.

## Introduction

The use of inhaled corticosteroids and long-acting β2-agonists has reduced asthma-related exacerbations [1]. However, some patients with bronchial asthma (BA) still suffer from symptoms in spite of the standard treatment. They are diagnosed as having severe asthma or refractory asthma [2,3], and their medical expenses are high. Their quality of life is reduced by the persistence of chronic airway obstruction and frequent exacerbations. Therefore, establishment of biomarkers reflecting the severity of BA and predicting exacerbations is of special importance.

Recently, reactive oxygen species (ROS) such as hydrogen peroxide have been attracting attention in a variety of respiratory disorders including asthma. Levels of ROS are an indicator of oxidative stress, and ROS are generated from inflammatory cells, which participate in the inflammatory response of BA [4]. However, the relationship between the degree of BA exacerbation in clinical settings and the degree of oxidative stress is not yet clear.

It has been reported that some biomarkers are useful in predicting the responses to certain agents among BA patients. For example, serum levels of periostin were associated with the response to anti-IL-13 antibody in severe asthma [5]. In neutrophilic asthma, sputum levels of interleukin-8 (IL-8) and IL-6 were associated with neutrophil numbers in the sputum and certain pulmonary function parameters [6,7]. These cytokines were reported to be released by airway epithelial cells and were suggested to play an important role in severe asthma [8]. However, few reports exist that show any clinical significance of the levels of ROS and the severity of asthma.

Therefore, the aim of this study was to measure oxidative stress among patients with BA and evaluate the roles of ROS in the clinical features of asthma.

## Methods

### Patients

We enrolled patients with BA from 2013 through 2015 at the Department of Respiratory Medicine, Kyorin University Hospital. The diagnosis of BA was made according to the criteria of the Global Initiative for Asthma (GINA) 2015. No patients had experienced any exacerbation of BA for more than 4 weeks prior to recruitment in this study. This study was approved by the Institutional Review Board of Kyorin University (No. 161), and all of the patients participated in the study upon providing written informed consent.

### Pulmonary function tests

Pulmonary function tests were performed using a SYSTEM 21^®^ device (MINATO MEDICAL SCIENCE CO., Osaka, Japan) according to the criteria of the American Thoracic Society (ATS)/European Respiratory Society and the Japanese Respiratory Society.

### Blood cell counts

Peripheral blood cell counts were performed on each patient.

### Measurement of fractional exhaled nitric oxide (FeNO) level

The FeNO level was measured using a NIOX MINO^®^ device (Aerocrine AB, Solna, Sweden) according to the manufacturer’s instructions and the ATS guidelines [9].

### Measurement of serum reactive oxygen metabolite (ROM) level

To assess oxidative stress, instead of measuring serum ROS directly, we measured serum ROM. The serum ROM level was measured with the d-ROMs test using the FREE carpe diem^®^ system (Diacron International, Grosseto, Italy) according to the manufacturer’s instructions. The principle of the d-ROMs test is to measure hydroperoxide using the Fenton reaction.

The units of this test are expressed as CARR U. One CARR U is equivalent to 0.08 mg/dl of hydrogen peroxide. The normal range of this test was established as 200–300 CARR U, and the borderline, low, middle, and high levels of this test were established as 301–320, 321–340, 341–400, and over 400 CARR U, respectively [10].

### Measurement of serum IL-8 and IL-6

Serum IL-8 and IL-6 were measured using an ELISA kit (R&D Systems, Minneapolis. MN).

### Glutathione S-transferase P1 (GSTP1) polymorphism genotyping

We identified exon 5 polymorphism (*Ile*105*Val*) of GSTP1 genotypes based on the report by Ishii et al. [11]. Three genotypes exist: AA (homozygous wild, *Ile*/*Ile*) type, AG (heterozygote, *Ile*/*Val*) type, and GG (homozygous mutant, *Val*/*Val*) type [12,13].

### Severe exacerbation

We determined whether the patients had a severe exacerbation of BA within 3 months after enrollment in this study. We defined severe exacerbation as having episodes of BA requiring hospitalization or an emergency room visit.

### Statistical analysis

All data are expressed as median (25^th^-75^th^ percentile) or number (%). Statistical analyses were performed using IBM SPSS Statistics version 23.0 (SPSS, Chicago, IL). Differences between groups were assessed using the Mann-Whitney U test or Kruskal-Wallis test. Categorical variables were assessed using the chi-square test or Fisher's exact test. A value of *p* < 0.05 was considered to be statistically significant. Correlation analyses were performed using Spearman’s correlation test. Receiver operating characteristic (ROC) curves were used to assess the discriminative power of serum ROM levels. The cutoff point for serum ROM level was determined as the minimum value of (1-sensitivity)^2^ + (1-specificity)^2^.

## Results

### Patient characteristics

We enrolled 110 patients with BA. The characteristics of patients are shown in Table 1. Their median age was 51 years, and 66 of the patients were non-smokers. Treatment steps of BA were staged according to the GINA criteria as Step 1 in four patients, Step 2 in 14 patients, Step 3 in 22 patients, Step 4 in 60 patients, and Step 5 in 10 patients. Symptoms of BA were controlled in 29.1%, partly controlled in 51.8%, and uncontrolled in 19.1% of the patients. The median serum ROM level was 345 CARR U, and the median FeNO value was 24 ppb.

**Table 1:** Patient Characteristics.

	Patients (N = 110)
Age, years	51 (43–64)
Female (%)	66 (60.0)
BMI, kg/m^2^	23.0 (20.8–27.6)
Non-smoker (%)	66 (60.0)
Ex- or current smoker (%)	44 (40.0)
Atopy (%)	11 (10.0)
Allergic rhinitis (%)	51 (46.4)
Chronic rhinosinusitis (%)	23 (20.9)
GINA treatment steps	
Step 1 (%)	4 (3.6)
Step 2 (%)	14 (12.7)
Step 3 (%)	22 (20.0)
Step 4 (%)	60 (54.5)
Step 5 (%)	10 (9.1)
GINA symptom control	
Well controlled	32 (29.1)
Partly controlled	57 (51.8)
Uncontrolled	21 (19.1)
Use of ICS (%)	100 (90.9)
Use of LABA (%)	87 (79.1)
Use of LTRA (%)	53 (48.2)
WBC, ×10^9^/L	6.5 (5.2–7.3)
Neutrophils, ×10^9^/L	3.8 (2.8–5.0)
Eosinophils, ×10^9^/L	0.2 (0.1–0.3)
IgE, IU/mL	182 (45–643)
IL-8, pg/mL	13.4 (10.2–16.9)
IL-6, pg/mL	1.1 (0.5–2.0)
CRP, mg/dL	0.10 (0.00–0.20)
Serum ROM levels, CARR U	345 (311–385)
VC, L	3.0 (2.5–3.9)
%VC, %	107.3 (94.0–114.2)
FVC, L	2.9 (2.4–3.8)
%FVC, %	97.1 (85.6–107.6)
FEV_1_, L	2.3 (1.7–2.9)
FEV_1_, % predicted	88.6 (74.1–103.6)
FEV_1_/FVC, %	75.5 (67.9–81.2)
FeNO, ppb	24 (16–43)
Exon 5 polymorphism of GSTP1 genotypes, AA/AG/GG/unknown	58/48/1/3

Data are shown as median (25^th^-75^th^ percentile) or number (%). BMI, body mass index; GINA, Global Initiative for Asthma; ICS, inhaled corticosteroid; LABA, long-acting β2-agonist; LTRA, leukotriene receptor antagonist; WBC, white blood cells; IgE, immunoglobulin E; IL-8, interleukin-8; IL-6, interleukin-6; CRP, C-reactive protein; ROM, reactive oxygen metabolite; VC, vital capacity; FVC, forced vital capacity; FEV_1_, forced expiratory volume in 1 second; FeNO, fractional exhaled nitric oxide; GSTP1, glutathione S-transferase P1.

### Relationship between serum ROM levels and other biomarkers

**Fig 1:**
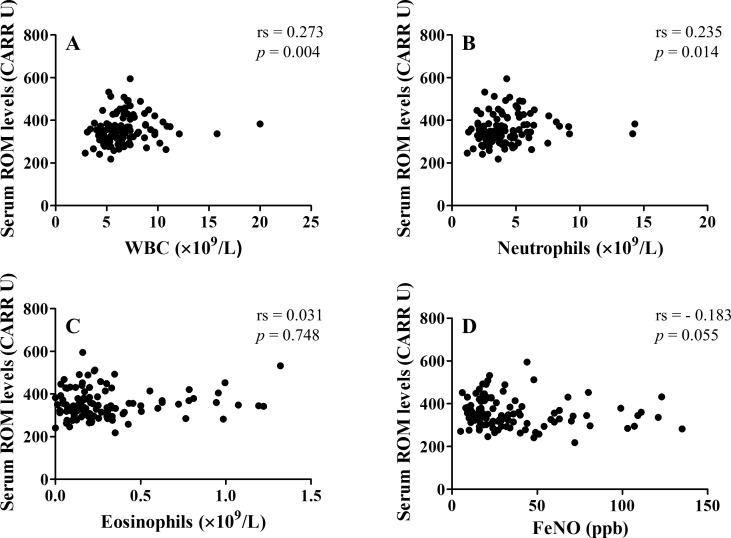
Correlation between serum reactive oxygen metabolite (ROM) levels and biomarkers. (A) Serum ROM levels and white blood cell (WBC) count. (B) Serum ROM levels and neutrophil count. (C) Serum ROM levels and eosinophil count. (D) Serum ROM levels and fractional exhaled nitric oxide (FeNO).

Serum ROM levels correlated with white blood cell (WBC) count (rs = 0.273, *p* = 0.004), neutrophil count (rs = 0.235, *p* = 0.014), and serum levels of C-reactive protein (CRP) (rs = 0.403, *p* < 0.001) and IL-6 (rs = 0.339, *p* < 0.001) but not with serum levels of IL-8 (Table 2, Fig 1A and 1B). Serum ROM levels did not correlate with eosinophil count or FeNO (Fig 1C and 1D).

**Table 2:** Correlation Between Serum ROM Levels and Biomarkers.

	Serum ROM levels	
	Correlation coefficients	*p* value
WBC	0.273	0.004
Neutrophils	0.235	0.014
Eosinophils	0.031	0.748
IgE	0.018	0.849
CRP	0.403	< 0.001
IL-8	0.037	0.703
IL-6	0.339	< 0.001
FeNO	- 0.183	0.055

ROM, reactive oxygen metabolite; WBC, white blood cells; IgE, immunoglobulin E; CRP, C-reactive protein; IL-8, interleukin-8; IL-6, interleukin-6; FeNO, fractional exhaled nitric oxide.

### Correlation between serum ROM levels/biomarkers and pulmonary function test results

**Fig 2:**
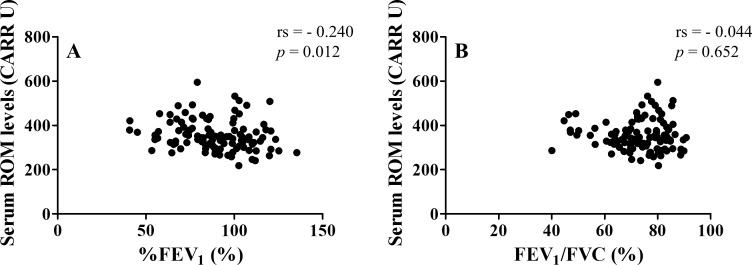
Correlation between serum reactive oxygen metabolite (ROM) levels and pulmonary function parameters. (A) Serum ROM levels and %FEV_1_. (B) Serum ROM levels and FEV_1_/FVC. %FEV_1_, percent predicted forced expiratory volume in 1 second; FVC, forced vital capacity.

Serum ROM levels negatively correlated with the percent predicted forced expiratory volume in 1 second (%FEV_1_) (rs = - 0.240, *p* = 0.012) (Table 3) (Fig 2A). However, there was no correlation between serum ROM levels and the FEV_1_/forced vital capacity (FVC) ratio (rs = - 0.044, *p* = 0.652) (Fig 2B). IL-8 negatively correlated with %FEV_1_ and FEV_1_/FVC (rs = - 0.362, *P* < 0.001 and rs = - 0.350, *p* < 0.001, respectively). WBC count, neutrophil count, eosinophil count, CRP, and FeNO did not correlate significantly with either %FEV_1_ or FEV_1_/FVC.

**Table 3:** Correlation Between Pulmonary Function Test Results and Biomarkers and Serum ROM Levels.

	%FEV_1_ (% predicted)		FEV_1_/FVC (%)	
	Correlation coefficients	*p* value	Correlation coefficients	*p* value
WBC	- 0.155	0.105	0.072	0.452
Neutrophils	- 0.123	0.200	0.067	0.490
Eosinophils	- 0.108	0.261	- 0.081	0.403
IgE	- 0.151	0.115	- 0.208	0.029
CRP	- 0.197	0.039	- 0.016	0.872
IL-8	- 0.362	< 0.001	- 0.350	< 0.001
IL-6	- 0.099	0.305	0.021	0.826
Serum ROM levels	- 0.240	0.012	- 0.044	0.562
FeNO	-0.033	0.732	- 0.081	0.400

FEV_1_, forced expiratory volume in 1 second; FVC, forced vital capacity; WBC, white blood cells; IgE, immunoglobulin E; CRP, C-reactive protein; IL-8, interleukin-8; IL-6, interleukin-6; ROM, reactive oxygen metabolite; FeNO, fractional exhaled nitric oxide.

### Relationship between serum ROM levels and GINA symptom control/treatment steps

We compared serum ROM levels between different groups of GINA symptom control levels and treatment steps. The serum ROM level of the well-controlled patients was 326 (295–370) CARR U. That of partly controlled patients was 345 (319–408) CARR U, and that of uncontrolled patients was 360 (313–407) CARR U. The ROM levels of the uncontrolled group were higher than those of the controlled group or partly controlled group but not significantly so (*p* = 0.183). Serum ROM levels were not significantly different between treatment steps (Step 1: 352 (251–457) CARR U, Step 2: 335 (294–357) CARR U, Step 3: 371 (312–433) CARR U, Step 4: 345 (309–379) CARR U, and Step 5: 364 (324–424) CARR U, *p* = 0.639).

### Biomarkers and serum ROM levels in exon 5 polymorphism of GSTP1 genotypes

In the 110 patients, AA type was the most frequent (n = 58), and only one patient had GG type. The GSTP1 genotypes were not identified in three patients. Among the tested biomarkers, only the serum levels of CRP were significantly different between AA type and AG type (0.10 [0.06–0.29] mg/dL vs. 0.09 [0.00–0.20] mg/dL, *p* = 0.042) (Table 4). The serum ROM levels were not significantly different between AA type and AG type (351 [319–412] CARR U vs. 339 [296–382] CARR U, *p* = 0.299).

**Table 4:** Biomarkers and Serum ROM Levels in Exon 5 Polymorphism of GSTP1 Genotypes.

GSTP1 genotypes	AA	AG	*p* value
WBC, ×10^9^/L	6.6 (5.4–7.3)	6.3 (5.2–7.6)	0.744
Neutrophils, ×10^9^/L	3.8 (2.9–5.0)	3.9 (2.8–5.1)	0.694
Eosinophils, ×10^9^/L	0.2 (0.1–0.3)	0.2 (0.1–0.3)	0.379
IgE, IU/mL	232 (45–833)	160 (58–490)	0.864
CRP, mg/dL	0.10 (0.06–0.29)	0.09 (0.00–0.20)	0.042
IL-8, pg/mL	13.4 (10.9–17.2)	12.9 (9.6–16.6)	0.386
IL-6, pg/mL	1.1 (0.5–2.4)	1.1 (0.5–1.9)	0.849
Serum ROM levels, CARR U	351 (319–412)	339 (296–382)	0.299
FeNO, ppb	23 (16–44)	24 (16–43)	0.851

Data are shown as median (25^th^-75^th^ percentile). GSTP1, glutathione S-transferase P1; WBC, white blood cells; IgE, immunoglobulin E; CRP, C-reactive protein; IL-8, interleukin-8; IL-6, interleukin-6; ROM, reactive oxygen metabolite; FeNO, fractional exhaled nitric oxide.

### Role of serum ROM levels in predicting exacerbations

**Fig 3:**
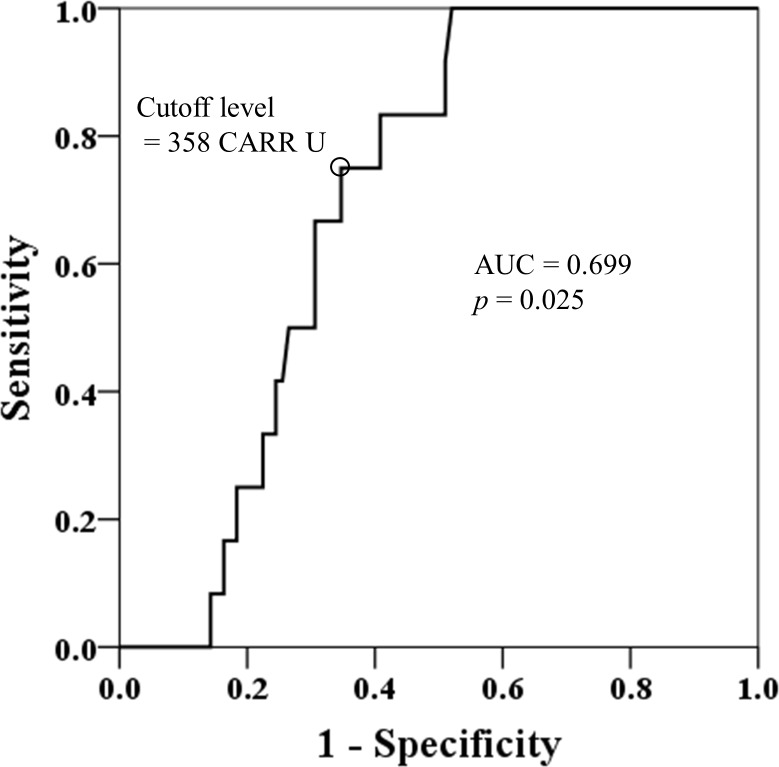
Receiver operating characteristic curve for serum reactive oxygen metabolite (ROM) levels associated with severe exacerbation in 3 months. The cutoff level was 358 CARR U. AUC, area under the curve.

During the study period, 12 episodes of severe exacerbation occurred in 12 patients within 3 months. The breakdown of GINA treatment levels was one patient with Step 2 treatment, seven with Step 4 treatment, and four with Step 5 treatment. Serum ROM levels were significantly higher in the patients who experienced severe exacerbation within 3 months than in the patients who did not (376 [352–414] CARR U vs. 339 [302–381] CARR U, *p* < 0.025) (Table 5). This was also the case for WBC count and neutrophil count between the two groups. In contrast, eosinophil count was significantly lower in the severe exacerbation group than in the non-severe exacerbation group. The ROC curve showed that serum ROM levels were associated with severe exacerbation at 3 months (area under the curve [AUC] 0.699, sensitivity 75.0%, specificity 65.3%, *p* = 0.025) (Fig 3). The cutoff level of 358 CARR U showed the highest discriminative power for the prediction of having severe exacerbation (odds ratio: 5.647, 95% confidence interval [CI]: 1.433–22.251).

**Table 5:** Biomarkers and Serum ROM Levels Between Groups With and Without Severe Exacerbation.

	Non-Sev-Ex group(N = 98)	Sev-Ex group(N = 12)	*p* value
Age, years	51 (44–64)	49 (33–71)	0.598
BMI, kg/m^2^	22.8 (20.6–26.9)	24.1 (21.4–30.1)	0.216
WBC, ×10^9^/L	6.3 (5.2–7.2)	10.1 (6.3–11.9)	0.002
Neutrophils, ×10^9^/L	3.7 (2.8–4.8)	8.3 (4.5–12.9)	0.001
Eosinophils, ×10^9^/L	0.2 (0.1–0.3)	0.1 (0.0–0.3)	0.013
IgE, IU/mL	173 (45–514)	228 (15–1403)	0.924
IL-8, pg/mL	13.3 (10.3–16.8)	14.9 (9.6–22.4)	0.673
IL-6, pg/mL	1.0 (0.5–1.7)	2.1 (0.7–3.5)	0.075
CRP, mg/dL	0.1 (0.0–0.2)	0.1 (0.0–0.5)	0.449
Serum ROM levels, CARR U	339 (302–381)	376 (352–414)	0.025
FeNO, ppb	25 (17–43)	16 (11–80)	0.146

Data are shown as median (25^th^-75^th^ percentile). Sev-Ex, severe exacerbation; BMI, body mass index; WBC, white blood cells; IgE, immunoglobulin E; IL-8, interleukin-8; IL-6, interleukin-6; CRP, C-reactive protein; ROM, reactive oxygen metabolite; FeNO, fractional exhaled nitric oxide.

## Discussion

We studied 110 patients with BA to determine the role of the oxidative stress biomarker serum ROM level in the clinical setting and management of BA. Serum ROM levels correlated with WBC count, neutrophil count, and CRP and IL-6 levels. In addition, serum ROM levels and IL-8 and CRP levels negatively correlated with %FEV_1_. These observations suggested that the serum ROM level might be a biomarker of neutrophilic asthma. We further found that serum ROM levels were significantly higher in patients who experienced severe exacerbation than in patients who did not. ROC analysis showed that ROM levels correlated significantly with the occurrence of severe exacerbation (AUC: 0.699, 95% CI: 0.597–0.801, *p* = 0.025). Therefore, ROM levels might be useful in predicting severe exacerbations of BA.

BA is characterized by airflow obstruction associated with chronic airway inflammation [14]. The majority of BA patients are controlled by standard anti-inflammatory therapy including inhaled corticosteroids, but a distinct group of patients shows a persistent decrease in FEV_1_/FVC and %FEV_1_ with frequent exacerbations, which require intensive care and large medical expenses [2]. The aims of clinically appropriate asthma management are both to control asthma symptoms and to reduce the risk of exacerbations. Conventional treatment has been largely based on the patient’s symptoms and lung function parameters [15]. For example, Li et al. reported that low respiratory function is a risk factor for BA exacerbations [16]. It is very important to assess the severity of BA accurately to provide the best treatment for each patient. Therefore, the establishment of more sophisticated biomarkers other than symptoms or respiratory function tests is of urgent importance.

In recent years, several biomarkers of BA have been reported for predicting exacerbations. Type-2 cytokine-driven eosinophilic airway inflammation tends to frequent exacerbations and a probable response to inhaled corticosteroids. In this context, the number of sputum eosinophils has been reported to be useful as both a marker of exacerbation risk and a biomarker for the adjustment of corticosteroid treatment [17]. Serum periostin is attracting attention as an inflammatory biomarker of the Th2 type, which predicts responses to anti-IL-13 antibody [18]. FeNO is also a biomarker of a Th2-type inflammation and is reported to have an association with eosinophilic inflammation and the clinical control of asthma [9]. However, FeNO is influenced by a variety of factors such as smoking and treatment including corticosteroids, and its measurement is often difficult during asthmatic attacks. Because a significant proportion of asthmatic patients shows distinct characteristics of neutrophilic airway inflammation [19], novel surrogate biomarkers for neutrophilic inflammation would appear to be necessary. IL-8 has been reported in sputum [6,20]; however, because stable asthma patients expectorate little sputum, it is difficult to use biomarkers from sputum.

Oxidative stresses are reported to play a crucial role in the pathogenesis of airways in BA. Some inflammatory cells such as neutrophils produce ROS [21]. Nadeem et al. reported that BA patients show increased superoxide generation from leukocytes [22]. In addition, a relationship between oxidative stress and respiratory functions has been shown. For instance, Jarjour and Calhoun reported an association between airway obstruction and superoxide production by airspace leukocytes [23]. Rhoden and Barnes showed that hydrogen peroxide induced contraction of the isolated trachea of guinea pigs [24]. Hulsmann et al. reported that oxidative damage to the airway epithelium may lead to hyperresponsiveness to inhaled stimuli [25]. These reports strongly suggest that oxidative stresses are closely involved in the pathogenesis of BA.

Recently, several reports have emphasized an important role of oxidative stress in the clinical control and exacerbation of BA. Suzuki et al. showed that serum ROM levels were significantly higher in patients with acute exacerbation of BA than in those with stable BA [26]. Foschino Barbaro and colleagues reported that plasma ROM levels were significantly higher in patients with BA than in healthy subjects [27]. Melillo et al. also showed that ROM levels in serum and exhaled breath condensate were higher in patients with chronic obstructive pulmonary disease than in healthy subjects [28].

In this study, we focused on oxidative stress and related markers in sera and studied their association with pulmonary function parameters and other biomarkers. Oxidative stress is a phenomenon that favors the oxidation reaction side because of a collapse of the balance between oxidation and antioxidation reactions [29]. We assessed the degree of oxidative stress as serum ROM level with the d-ROMs test using the FREE carpe diem^®^ system, which enables measurement of the concentration of hydroperoxides, one of the ROMs according to the Fenton reaction, within few minutes in a single machine. Oxidative stress measured by this system was associated with several diseases such as coronary artery disease and hepatocellular carcinoma [30,31].

In our patients with BA, we initially found the median serum ROM levels to be 345 CARR U, higher than the normal range (200–300 CARR U), which was established based on the measurement of 4547 healthy subjects [10]. This result suggested that patients with BA have higher stress levels than healthy subjects. Furthermore, we found that the serum ROM levels correlated with WBC, neutrophil numbers, and CRP and IL-6 levels. In addition, serum ROM levels and IL-8 and CRP levels negatively correlated with %FEV_1_. These observations suggested that the serum ROM level might be a biomarker of neutrophilic inflammation and the severity of asthma. We further found that serum ROM levels were significantly higher in patients who experienced severe exacerbation than in patients who did not. ROC analysis showed that ROM levels correlated significantly with the occurrence of severe exacerbation. Therefore, ROM levels might reflect neutrophilic inflammation and be useful in predicting severe exacerbations of BA. The d-ROMs test used in the current investigation has several advantages for clinical practice: the test is easy to perform, takes only several minutes, and requires as little as 20 μL of serum or plasma.

We further analyzed the exon 5 polymorphism of GSTP1 genotypes and studied its influence on serum ROM levels. GSTP1 genotypes have been reported to play a pivotal role in defense mechanisms against oxidative stress [32]. Several reports showed that GSTP1 genotypes were associated with asthma [33,34]. In a Taiwanese report, AA type was linked to a significantly increased risk of physician-diagnosed asthma (adjusted odds ratio: 1.94, 95% CI: 1.08–3.59) [35]. In our study, serum ROM levels of the AA type were higher than those of the AG type, but the difference was not significant. Further study with more cases is necessary to clarify the importance of polymorphisms of GSTP1 genes in the severity and risk of exacerbations of asthmatic patients.

This study has several limitations. The number of studied patients was relatively small, and therefore, the number of patients having exacerbation was also small, which precluded multivariate analysis. In addition, we could not repeatedly measure the ROM levels during the study period, nor could we measure them at the time of exacerbation and amelioration. Therefore, further research is necessary to clarify the real-world usefulness of this novel biomarker in clinical practice.

In conclusion, we found serum ROM levels to be significantly higher in patients with severe exacerbation of BA than in those with non-severe exacerbation. The serum ROM level might be a useful biomarker to predict severe BA exacerbation by use of a rapid and easy-to-perform clinical test.
