# Correction: Serum Reactive Oxygen Metabolite Levels Predict Severe Exacerbations of Asthma

**DOI:** 10.1371/journal.pone.0168220

**Published:** 2016-12-09

**Authors:** The PLOS ONE Staff

## Notice of Republication

This article was republished on November 8, 2016, as a Supporting Information file was published in error. The publisher apologizes for the error. Please download this article again to view the correct version.
